# Cognitive and preference disparities of Chinese consumers regarding the disclosure of unsafe food recall information

**DOI:** 10.3389/fpubh.2024.1467518

**Published:** 2024-12-09

**Authors:** Taiping Li, Xiaohui Jin, Daocai Zhu

**Affiliations:** ^1^College of Economics and Management, Nanjing Agricultural University, Nanjing, China; ^2^School of Economics, Anhui University of Finance and Economics, Bengbu, China

**Keywords:** unsafe food recall, information disclosure, cognitive dissonance, altruism, bystander effect

## Abstract

**Introduction:**

Information disclosure is important in promoting unsafe food recalls and reducing potential food safety risks. However, the governance of unsafe food recall information in China is distorted, leading to cognitive dissonance in Chinese consumers’ perceptions of unsafe food recall information. Focusing on consumers’ search and cognitive costs, this study suggests that market regulators should proactively and fully disclose unsafe food recall information to satisfy consumers’ needs and preferences for recall information, thereby optimizing consumer perceptions and facilitating the improvement of the information governance system for unsafe food recalls.

**Methods:**

This study administered a survey via a discrete choice experiment to obtain data from 1,010 consumers in China and employed multiple linear regression (MLR) to analyze the overall cognition and preferences of consumers regarding food recall information and identify differences in cognition and preferences regarding unsafe food recall information.

**Results:**

Chinese consumers experience cognitive dissonance regarding food recall information, and their utility can be improved through disclosure. They expressed preferences for recall information about food shops and distribution markets, more visualized hazard content, and new media presentations. Those who had purchased unsafe food, families with pregnant women or children, and those with more education were more concerned about recall information. Consumers’ information preferences also show a bystander mentality; however, consumers with higher educational levels are more altruistic.

**Discussion:**

The results suggest that personalized, intuitive, and cognitively matched recall information can reduce consumers’ search and cognitive costs and increase their utility. This finding provides a reference and practical basis for establishing a food safety information governance system in China.

## 1 Introduction

**Figure 1:**
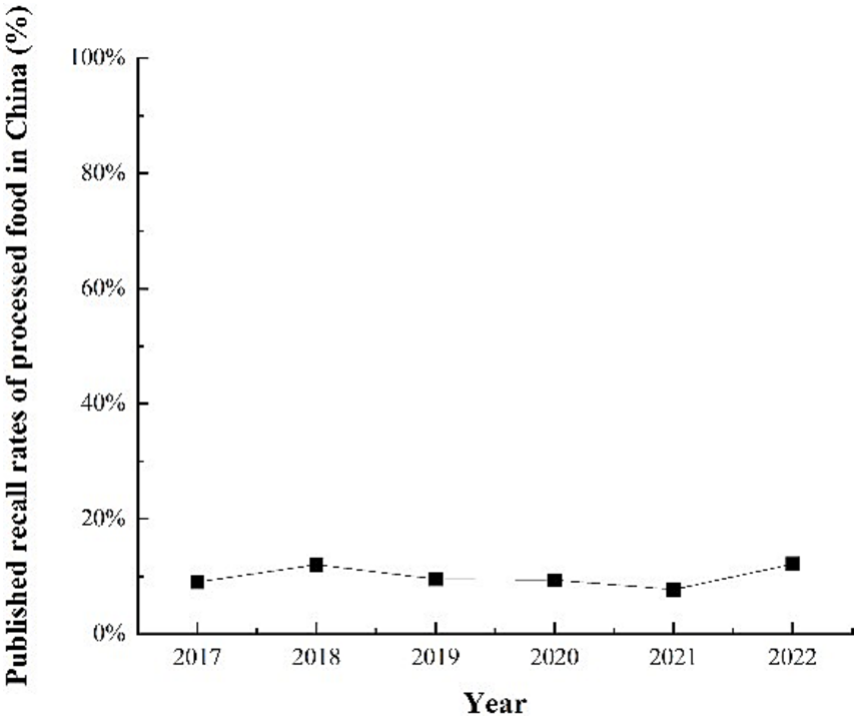
The average rate of food recalls publicized by Chinese province, 2017–2022.

**Figure 2:**
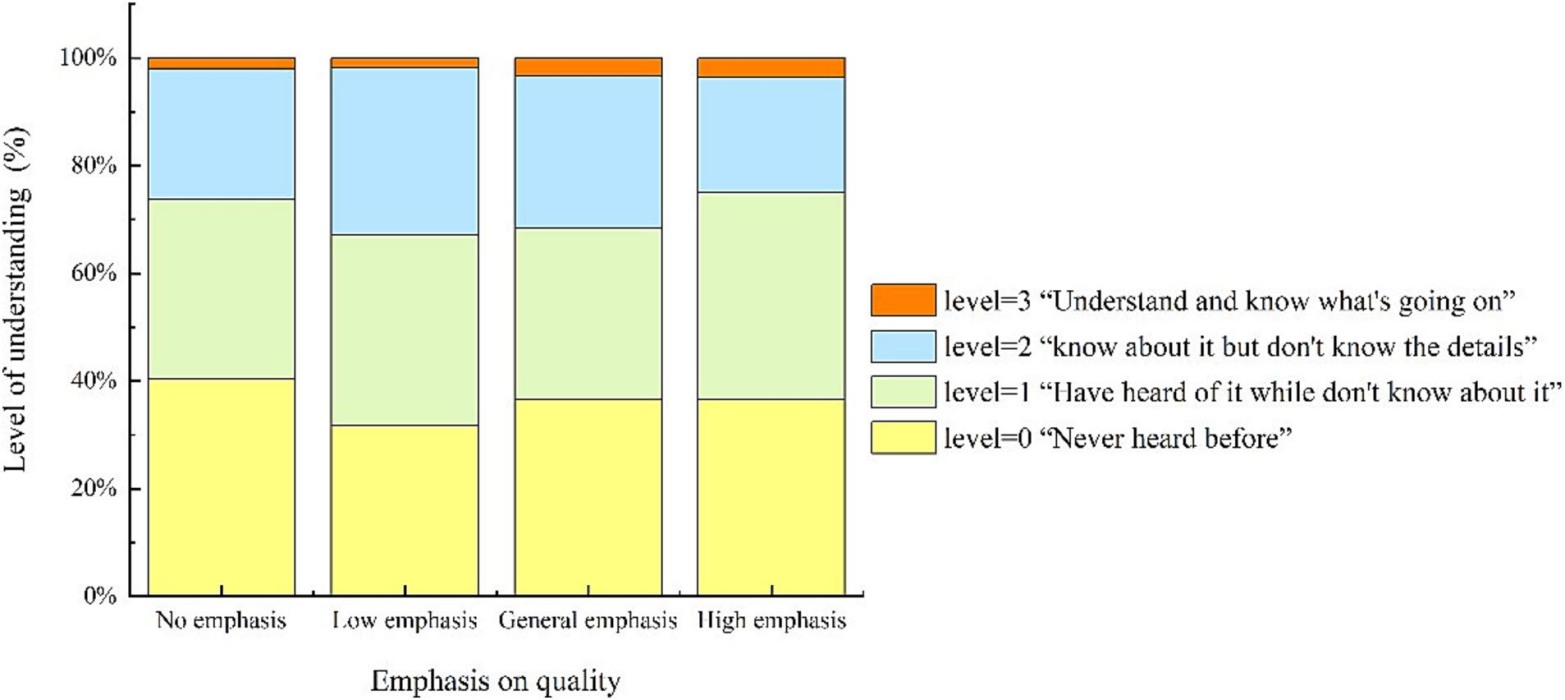
Proportion emphasizing quality by level of knowledge of food recall information.

China’s food safety governance faces challenges (1). Information asymmetry in the food industry and the resulting cognitive dissonance among consumers have been a longstanding significant concern in the public health field (2–4). Between 2008 and 2019, more than 430,000 food safety incidents occurred in China (5), impacting the health and safety of the Chinese people. However, the current situation indicates that China’s recalls of unsafe foods remain relatively low. Based on data on unsafe food incidents at the provincial level in mainland China from 2017 to 2022, the average recall rate was estimated to be approximately 7–12%^1^ (Figure 1). As unrecalled food poses potential food safety risks, since 2015, the Chinese government has revised food recall regulations to address unsafe food, the primary goal of which is to retrieve unsafe food, for which food enterprises are responsible. The new regulations emphasize information disclosure to allow recall disclosure to play an important role in the information governance of unsafe food. The regulations require the State Administration for Market Regulation (SAMR) and local market regulators to order food companies to carry out recalls at three levels based on the degree of impact and require food companies to inform sellers and consumers of the production batch, the reason for the recall, and the scope of the region, and to stop consuming unsafe food. Regulatory authorities publish risk warnings, verifications, and disposal information on their official websites. However, the results in Figure 1 show that information governance policies are dysfunctional. In 2023, the survey team’s findings indicated that consumers rarely had access to unsafe food recall information (see Figure 2). What prevents consumers from receiving and understanding the recall information?

Food companies, the bearers of unsafe food recalls, have little incentive to disclose such information. Many studies of food information disclosure suggest that food products have strong “trustworthy” characteristics (6), making it difficult to verify the quality of agricultural products (7). For consumers, food safety is often an afterthought, creating a significant information asymmetry among consumers, food producers, and distributors throughout the production, processing, distribution, and consumption processes (8), which facilitates opportunistic behavior by food producers (9–11). To address information asymmetry, consumers can rely only on nutritional information provided by food companies (12), food labels (13), or quality safety certifications provided by third-party certification bodies (14). However, this entails the risk of adverse selection, because consumer behavior and attitudes toward food and labels encourage food companies to convey positive information voluntarily (15). Contrariwise, food companies are unwilling to disclose negative food safety information such as food recalls, which have a “contagious effect” (16). Such behavior can cause widespread consumer behavior changes in the market (17). For example, the recall of beef with *E. coli* has reduced consumer demand for ground beef (18, 19), and the recall of packaged spinach caused by *E. coli* has led to a significant decline in spinach sales (20). Corporate recalls can affect consumers’ willingness to buy and their perceptions of food safety (21). It is not appropriate to place too much responsibility on companies for disclosure. This implies that consumers must pay higher search costs to obtain information on corporate recalls.

Moreover, news media coverage of food incidents is selective. The news media tend to prioritize major events and are biased because of underlying political and socioeconomic views (22). Owing to the clustering behavior of social information dissemination (23), exposure by the news media makes consumers prone to overreaction in the food industry (24–26). Many studies have shown that consumers in developing countries have a lower level of awareness of the relationship between production methods and food safety than those in developed countries (27, 28), making them more susceptible to food safety panics (29). In China, food safety panics occur frequently because of consumers’ low awareness of food safety issues. For example, during the 2003 SARS outbreak in China, distorted information transmission caused a food panic in Guangzhou Province (30). The recall of dairy products in China triggered a food safety panic, significantly changing consumer purchasing behavior (31). Consequently, news media coverage of food recalls fails to meet consumers’ daily information needs and can cause cognitive distortions.

The critical role of digital platforms and social media in disseminating food safety information is increasingly evident. Early research by Thackeray et al. (32), who conducted a cross-sectional study of state public health departments, indicated that social media has gradually emerged as an essential tool for public health agencies to communicate health information. However, this study also highlighted the need to enhance public interaction to strengthen public health communication. With the widespread adoption of new media technologies, social media platforms—characterized by rapid dissemination, accessibility, and interactivity—have played a pivotal role in communicating food risks and benefits (33). Increasing consumer trust in social media has heightened risk perception regarding food safety (34), with more consumers relying on online platforms for food risk information (35–37). Furthermore, Avelino et al. (38) highlighted the effectiveness of platforms like Facebook and Instagram in promoting healthy eating and nutrition education, particularly among low-income groups, through their broad user coverage and engagement levels.

It is also important to combine information disclosure in the food industry with government regulations to maximize its impact (39). Regulatory authorities can leverage information disclosure as a low-cost regulatory tool (40–42), particularly in food safety (43). For instance, the United States has established a comprehensive food recall information exchange system. Through FDA and FSIS websites, the public receives detailed information on risk levels, product specifics, potential contamination, and recall volumes. Additionally, the National Outbreak Reporting System (NORS) records and monitors foodborne illnesses and disseminates health risk information to the public (44, 45). Similarly, the European Union’s food safety risk communication system excels in information dissemination, data collection, and public communication (46). Supported by transparent and standardized traceability mechanisms, these systems facilitate effective food recall and risk management (47). In contrast, China faces significant challenges due to the lack of transparency in its current food safety governance framework (2). The SARS epidemic made Chinese residents aware of the importance of citizens’ right to know and of government information disclosure (48). In particular, the greatest demand for food safety among Chinese consumers is for the disclosure of food safety information (49), which is a prerequisite for narrowing the gap between Chinese food safety information and consumer cognition (50). Figure 1 shows that China’s food recall program has not achieved its desired results. This finding suggests that information governance has not played a significant role in recall systems. Tang et al. (51) found that Chinese food information disclosure was not well combined with the recall system. The inefficiency of the regulator in information governance is demonstrated by the insufficient transmission of information, as well as the failure to reverse the bias in consumer perceptions of risk. A survey of Chinese residents found that less than one-tenth of the population could access such food safety-related information (3). There is also a divergence between consumers’ subjective risk perceptions and objective food safety conditions (4). Researchers believe that the Chinese government should correctly guide consumers through the timely and objective publication of food quality test results and strengthening food safety publicity (52). Faced with food safety risks, China’s food regulatory authorities should prioritize consumer rights and explain and convey risk information such as harmful foods and foods unsuitable for specific populations (53).

Extensive research has examined the actors responsible for disclosing food recall information and the effectiveness of such disclosures, particularly in addressing the challenges of information governance faced by the Chinese government. These studies have made significant contributions to these fields. However, several questions remain unanswered: How can the Chinese government achieve complete disclosure of food recall information asymmetry? How can consumer demand for recall information be satisfied to prevent cognitive dissonance? What heterogeneous factors influence consumer information needs? These critical issues require further in-depth exploration.

In China, the government is the most trusted institution among consumers. Consequently, in situations where food companies are reluctant to disclose information or news media coverage is selective, government disclosure becomes a critical channel for consumers to access food recall information. However, does the information disclosed by the Chinese government regarding unsafe food truly meet consumer needs? If not, what are consumer’s preferences for such information? Furthermore, how do individual characteristics, household factors, and, particularly, the level of education shape consumers’ perceptions of unsafe food recall information? The study seeks to address these key questions and research objectives. This study explores the following issues: First, it analyzes consumer perceptions of current unsafe food recall information based on field research data. Second, information that meets consumers’ needs and perceptions is analyzed from the perspectives of search cost and cognitive cost, and it is shown that consumers can gain utility growth through unsafe food recall information, which is verified by the results of the choice experiment method. Third, the existence of heterogeneity in consumers’ preferences for unsafe food recall information across different characteristic groups and the reasons behind this are analyzed. Finally, the role of education in information cognition and demand is discussed, providing a basis for guiding food regulators to scientifically disclose information and communicate food safety risks.

## 2 Conceptual framework and hypotheses development

### 2.1 Unsafe food recall disclosure and cognitive dissonance

Festinger (54) demonstrated that cognitive dissonance arises from inconsistencies between attitudes and behaviors. Consumers’ cognition is typically influenced by the information they encounter (55). In China, food safety panics occur frequently because of consumers’ low awareness of food safety issues (30, 31). And only a small percentage of the population could access this food safety information (3). Research on the impact of African swine fever on Chinese pork consumption also suggests that Chinese consumers have a low perception of food safety risks (56). Accordingly, we propose the following hypothesis:

*Hypothesis* 1: Consumers have cognitive dissonance about unsafe food recall information.

### 2.2 Search, cognition, and unsafe food recall information preferences

Consumers are limited by their cognitive ability and limited information in their ability to understand all information accurately and display limited rationality in their decision-making processes (57). Stigler (58) argued that with information asymmetry, people need to pay costs, such as fees, time, and effort, to obtain and process information. Zenon et al. (59) examined the cognitive cost of information, and proposed that the complexity of information and the need to process it affect people’s subjective perceptions of information. Therefore, we incorporate search and cognitive costs into consumer utility functions.

Assuming that the consumer’s utility function is *U*, consumers gain safety benefits by avoiding food safety risks when they are informed of an unsafe food recall *V*, and consumers have search costs $C_{S}$ and cognitive costs $C_{C}$ for unsafe food recall information. θ dictates the extent of the impact of negative news, θ ∈[0, 1]. *ν* and *μ* are coefficients, where ν indicates the level of consumer effort in searching for unsafe food recall information; larger *ν* means more $C_{S}$ , so $C_{S}$ is a is a monotonically increasing function of *ν*. *μ* denotes the level of cognitive ability of the unsafe food recall information, where larger *μ* means that the $C_{C}$ paid to understand the information is lower; thus, *μ* and $C_{C}$ are inversely related functions. Consumers are more likely to give up the search for unsafe food recall information as *ν* increases, and the θ they obtain decreases, so ν is inversely related to θ . When *μ* is higher, consumers better understand what the recall message conveys and the higher the degree of negative impact they receive, so *μ* and *θ* are positively related. For analysis, we construct θ as a one-time linear function, where the implicit condition is that the search behavior precedes the cognitive behavior; if a consumer has a low cognitive ability but requires a high level of effort to produce utility, then that consumer will not engage in the search behavior and θ will be meaningless. Based on the above discussion, this paper constructs Equations 1 and 2 to represent *U*_max_ and constraints.


(1) $$U_{\text{max}}=\theta .V-C_{S}-C_{C}$$

(2) $$s.t.\theta \left(\nu ,\mu \right)=-\nu +\mu ,\;0\leq \nu \leq 1;0\leq \mu \leq 1.$$

With the above constraints, we construct a Lagrangian function *L*. Which is expressed by Equation 3:


(3) $$L=\theta .V-C_{S}-C_{C}+\lambda .\left(\theta +\nu -\mu \right)$$

We find the first-order derivatives of θ , λ, ν, and *μ* in the above equation. Which is expressed by Equations 4–7:


(4) $$\frac{\partial U}{\partial \theta }=\frac{\partial }{\partial \theta }\left[\theta .V+\lambda .\left(\theta +\nu -\mu \right)\right]=V+\lambda =0$$

(5) $$\frac{\partial U}{\partial \lambda }=\frac{\partial }{\partial \lambda }\left[\theta +\nu -\mu \right]=\theta +\nu -\mu =0$$

(6) $$\begin{array}{c} \frac{\partial U}{\partial \nu }=\frac{\partial }{\partial \nu }\left[\theta .V-\frac{\partial C_{S}}{\partial \nu }+\lambda .\left(\theta +1\right)\right]=V.\frac{\partial \theta }{\partial \nu }-\frac{\partial C_{S}}{\partial \nu }+\lambda .\left(\frac{\partial \theta }{\partial \nu }+1\right)\\ =-V-\frac{\partial C_{S}}{\partial \nu }=0 \end{array}$$

(7) $$\begin{array}{c} \frac{\partial U}{\partial \mu }=\frac{\partial }{\partial \mu }\left[\theta .V-\frac{\partial C_{c}}{\partial \mu }+\lambda .\left(\theta -1\right)\right]=V.\frac{\partial \theta }{\partial \mu }-\frac{\partial C_{c}}{\partial \mu }+\lambda .\left(\frac{\partial \theta }{\partial \mu }-1\right)\\ =V-\frac{\partial C_{c}}{\partial \mu }=0 \end{array}$$

As $\frac{\partial C_{S}}{\partial \nu }>$ 0, $\frac{\partial U}{\partial \nu }=-V-\frac{\partial C_{S}}{\partial \nu }<0.$ As $\frac{\partial U}{\partial \nu }<$ 0, an increase in ν decreases *U*. Therefore, consumers tend to reduce the level of effort to maximize utility. In the same way, it is possible to show that $\frac{\partial C_{c}}{\partial \mu }<$ 0, $\frac{\partial U}{\partial \mu }$ = $V-\frac{\partial C_{c}}{\partial \mu }>$ 0. As $\frac{\partial U}{\partial \mu }>$ 0, increasing cognitive ability ν will increase total *U*. Therefore, consumers tend to increase their cognitive abilities to maximize their utility.

In this study, consumers’ effort and cognitive ability for unsafe food recall information were related to the intensity of personalization, professionalism, and intuitiveness of the information itself. Al-Bahrani (60) argues that researchers should emphasize the relevance and potential benefits of information to better communicate effectively with social audiences, while lay people have difficulty in grasping complex concepts and specialized outcomes; moreover, the use of clear and concise language, visual aids such as icons or infographics, and examples, without compromising on accuracy, can appeal to and motivate people to understand and appreciate the results. Yu et al. (61) argued that innovative new media can more effectively convey information visually than traditional media. Accordingly, we propose the following hypotheses:

*Hypothesis* 2a: Consumers’ access to information on unsafe food recalls can lead to an increase in their own utility.

*Hypothesis* 2b: Consumers tend to choose information that has a high search cost, such as “*Store Quantity*,” or a low cognitive cost, such as “*Hazardous substance*” and “*Video style*.”

### 2.3 Altruism, the bystander effect, and unsafe food recall information preference

Hallman et al. (62) suggested that consumers who have fallen ill from consuming unsafe food or whose friends or family members have had such experiences may be more vigilant and concerned about food safety issues. In such cases, if products from a certain brand are recalled, these consumers may receive the news more quickly and take appropriate actions, such as not purchasing products from that brand or promptly checking whether they have consumed the recalled products. If consumers have not experienced similar incidents, they may lack sufficient awareness and vigilance regarding food safety issues and may even ignore recall information (63, 64). This difference may be attributed to the “Bystander effect,” as exemplified by the parable of the “Good Samaritan” in Christian culture and the Chinese proverb “It’s none of my business” (65, 66). Many scholars (67–69) have argued that Chinese residents exhibit a collective consciousness. This collective consciousness, along with altruism, is a key component of Chinese moral values. Similar conclusions were drawn in studies of Western populations by Van (70) and Persson and Petri (71), who suggested that increased emotional empathy enhances altruistic values. Social norms can help cultivate children’s sense of fairness and generosity (72). Children or pregnant women need the care of their families, and families that take on caregiving roles are more empathetic (73).People develop a sense of collective identity during the empathy process, leading to altruistic behaviors (74, 75). Based on this argument, we propose the following hypotheses:

*Hypothesis* 3a: Consumers who have experienced unsafe food are able to derive more utility from unsafe food recall information than consumers who have not experienced unsafe food, prefer information that is relevant to them, and focus on socially relevant recall information owing to altruism arising from empathy.

*Hypothesis* 3b: Families with pregnant women or children are more likely to empathize and pay more attention to information such as “*Number of food products recalled*” and “*Handling of recalled food*,” as2 well as socially relevant recall information such as “*Sales quantity*.”

### 2.4 Education and unsafe food recall information preferences

Education enhances cognitive abilities by improving skills, and those with higher levels of education tend to show greater cognitive functioning throughout adulthood (76). Extensive research has also revealed a positive association between educational level and behaviors such as charitable giving and volunteering, possibly because people with higher levels of education have more altruistic tendencies (77). For example, increasing the years of education positively affects both formal and informal volunteering (78). Individuals who have completed more than 7 years of schooling are more likely to be volunteers and donors (79). Those with higher education donate 77% more to charity than those with primary education (80). A similar pattern exists for Chinese residents, with rural households in some regions individually donating more as the number of years of education increases (81). Three rounds of data from the China Labor Force Dynamics Survey show that the likelihood of sustained household giving increases as the level of household education increases (82). These studies show that well-educated people have a greater cognitive ability to recognize the public interest of society and that the knowledge, moral literacy, and competence gained through education are stronger. Based on this argument, we propose the following hypothesis:

*Hypothesis* 4: Higher education improves altruistic behavior; consumers with higher education pay more attention to socially relevant recall information such as “*Sales quantity*” and “*Handling of recalled food*” than those without higher education; consumers with higher education have better cognitive abilities and are more receptive to visual “*Hazardous substance*” and new media expressions.

Based on the above analyses, consumers theoretically suffer from cognitive dissonance in the current unsafe food recall disclosure. Thus, improving information disclosure to reduce consumers’ search and cognitive costs can increase consumers’ utility. Differences in information preferences among consumer groups with different characteristics demonstrate the bystander effect and altruism. Education can play a role in cognitive ability and moral literacy, allowing consumers to show greater altruism in their information preferences.

**Figure 3:**
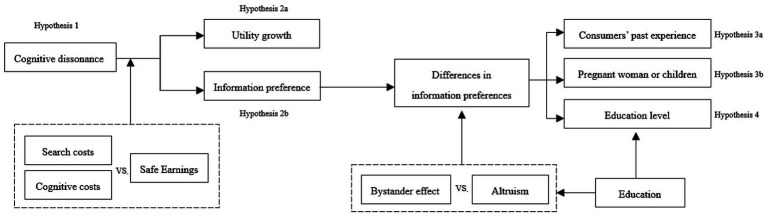
Research framework.

The research framework is shown in Figure 3.

## 3 Materials and methods

### 3.1 Data source

Data were collected through field surveys conducted between 2022 and 2023. A pre-survey sample of 160 questionnaires from Jiangsu and Anhui Provinces was used to determine the final questionnaire and selection set. The formal survey, employing a random sampling method, was conducted from April to July 2023, using face-to-face interviews with one person per household. In some remote western areas, data collection was assisted by recruited surveyors who were trained and supervised in real time by the researchers via phone and WeChat. The questionnaires were cross-checked for quality assurance and labeled, and ineligible questionnaires were discarded. The study covered the Xinjiang Uygur Autonomous Region and Anhui and Jiangsu Provinces, representing western, central, and eastern China, with 300 questionnaires distributed in each region. During the survey, data were collected sporadically from Ningxia, Sichuan, Yunnan, Guangdong, Zhejiang, and other areas, and supplemented with scattered samples from urban areas provided by the researchers’ relatives, friends, and classmates. A total of 1,192 questionnaires were distributed in the formal survey. After removing incomplete or inaccurate questionnaires, 1,010 valid samples were obtained, for a validity rate of 84.73%, ensuring the broad coverage and universality of the sample.

The Chinese government attaches great importance to food safety issues and has revised and improved relevant laws on food recalls multiple times. Once food testing results indicate unsafe conditions, market supervision authorities are responsible for promptly issuing unsafe food recall information to convey food safety risk information to consumers. However, Chinese consumers face low levels of acquisition and cognition of food safety risks (3, 56). Therefore, it is necessary to analyze whether cognitive dissonance exists in consumers’ understanding of unsafe food recall information and their actual preferences to enable market supervision departments to improve the disclosure of unsafe food recall information and compel recalls of unsafe food products.

### 3.2 Correlated variable selection and measurement

#### 3.2.1 Emphasis on quality

This is an important foundational variable in this study. If consumers do not place high emphasis on food quality, they will not care about it. Their survey results were not carefully considered but rather randomly chosen, and the research findings based on this may not convince others. This study follows Nie (83) in assessing consumers’ emphasis on food quality, which we measured by asking consumers, “Which of the following pieces of information do you value most when buying food? Please select and rank according to your purchasing habits, or you can choose not to answer.” Options include taste, price, quality, brand, manufacturer, shelf life, packaging, and certification, and the score is assigned based on the ranking of the “quality” option by consumers. If consumers do not choose the “quality” option, it is considered not important and scored 0 points; if consumers rank “quality” in *n*th place out of *N* choices, the score is calculated as 10 − 9*n*/*N* (assigning scores from 0 to 9). Table 1 shows that participants’ average emphasis on the quality score was 5.020, indicating that their emphasis on food quality was moderate.

**Table 1:** Sociodemographic characteristics of the sample.

Variables	Description and assigned values	Mean	SD
Sex	Man = 1; Woman = 0	0.443	0.497
Experience	Whether have bought unsafe food Yes = 1; No = 0	0.560	0.496
Pregnant woman or children	The presence of pregnant women and children in the household Yes = 1; No = 0	0.322	0.467
Educational experience	High school diploma or less = 1; High school or technical secondary school = 2; Regular college or junior college = 3; Postgraduate and above = 4	2.842	0.772
Income (per month)	Below ¥3,000 = 1; ¥3,000–¥5,000 = 2; ¥5,001–¥8,000 = 3; ¥8,001–¥10,000 = 4; ¥10,001–¥15,000 = 5; ¥15,001–¥20,000 = 6; ¥20,001–¥30,000 = 7; more than ¥30,000 = 8	3.941	1.900
Emphasis on quality	The level of attention to quality Score from 0 to 9	5.020	3.103
Levels of understanding	Never heard of it = 0	0.949	0.856
	Have heard of it but do not know about it = 1		
	Know about it but do not know the details = 2		
	Understand it and know what is going on = 3		

#### 3.2.2 Levels of understanding

This is also an important foundational variable in this study. If consumers have a high level of understanding of unsafe food recall information and their understanding aligns completely with the intentions of unsafe food recall information disseminators, there is no cognitive dissonance and no need for further research. This study measured consumers’ understanding of unsafe food recall information by asking, “Do you know about food recalls?” and assigning scores based on the consumers’ descriptions of this type of information during the interviews (scored from 0 to 3; see Table 1 for details). Table 1 shows that participants’ average understanding score of this information is 0.949, indicating that they have a low overall level of understanding of food recalls, basically at the level of not understanding.

This study collected information such as gender, presence of pregnant women or children in the household, educational background, and income of the respondents and elicited whether participants had consumed unsafe food, their level of emphasis on food quality, and their understanding of unsafe food recall information. The demographic characteristics of the survey participants and key variables are summarized in Table 1.

### 3.3 Experiment design and characteristics

Since 2016, market supervision authorities at all levels in China have announced all food sampling results and comprehensive unsafe food recall information on their official websites. We collected recall announcements regarding unsafe food products from 31 provincial-level market supervision and administration departments in mainland China. The analysis revealed that after detecting unsafe food products through sampling, the content and presentation of the unsafe food recall information announced by local market supervision authorities were not entirely consistent. In particular, in cases of severe quality violations or when the food is widely consumed, the news media may report and track certain batches of unsatisfactory sampled food products. This real-world scenario provides the practical and policy simulation background used in this study. Given that unsafe food recall information, which is crucial negative information in food safety, has not been widely disseminated to consumers, there is a need to organize, analyze, and redesign the relevant elements of unsafe food recall information that consumers are concerned about. This study pre-collected and screened the content of existing information announcements, combined with pre-survey data on unsafe food recall information that consumers believe needs to be understood, to form selectable elements for designing choice sets.

#### 3.3.1 Attribute selection

In the first pre-survey phase, we invited 30 primary food purchasers from diverse backgrounds for face-to-face interviews lasting approximately 20–30 min each to understand the key attributes of unsafe food recall information that consumers prefer, allowing them to verbally express the information they desired without providing leading options to prevent distortion of the interview results. This information is classified into three categories (Table 2). For instance, phrases like “How many stores are still selling unsafe food after inspection?” were classified as “*Number of food products recalled*”; “What items were found to be unsafe during inspection and do they pose significant health risks?” as “*Reasons for food recall*”; and “What happens after food is recalled?” as “*Handling of recalled food*.”

**Table 2:** Selected attributes and levels for experimental design.

Attributes	Attributes levels
Number of food products recalled	Sales quantity Stores quantity Production quantity*
Reasons for food recalls	Hazardous substance Disqualified item*
Handling of recalled food	Video style Image style Text style*
Information fitness(*IF*)	0, 25, 50, 75, 100%

*indicates that the variable is a reference level.

#### 3.3.2 Level of selection

In the second phase of preliminary research, we randomly selected 80 consumers on the street for face-to-face interactions in which they described and selected details of the unsafe food recall information provided. We sought to identify the description and selection most commonly chosen by consumers, while ensuring that the information levels under the same attribute were mutually exclusive. Regarding the number of unsafe food recalled, we translated the colloquial expression “How many such foods are still being sold in the market?” into “*Sales Quantity*”; “How many unsafe foods are there near my home?” into “*Store Quantity*”; and “How many unsafe foods has this company produced?” into “*Production Quantity*.” Concerning the reason for the recall, authentic official announcements presented two forms for testers to choose from: One used text to indicate the hazardous substances found in the food and the degree of harm to humans, referred to as “*Hazardous substance*”; the other used specific numerical values to indicate the items tested in the food, standard levels, and detected levels, known as “*Disqualified item*.” Regarding the handling of recalled unsafe food, the investigators showed consumers several announcement formats that appeared on official websites or in the media in different regions. The “*Text style*” displayed the legal provisions and procedures for handling unsafe food without showing the results of the handling process. The “*Image style*” showed pictures of the handling process, indicating that the food had been destroyed, without displaying the legal provisions and procedures for handling. The “*Video style*” showed short videos of the food handling process without displaying the legal provisions for handling. These three methods of handling unsafe foods differ in their contents (see Table 2).

#### 3.3.3 Constraint selection

The disclosure of unsafe food recall information is a public good provided to consumers by the Chinese government. First, we have to make sure that the information provided is complete, so we have to avoid the appearance of “*No-reference information*” when setting the horizontal level. Second, public goods are rights that taxpayers should enjoy, and the government’s disclosure of food safety-related information enhances public welfare and is therefore mandatory. As this study simulates policy implementation scenarios to elicit consumer preferences, constraints should not appear. In this study, the “information fitness” of unsafe food recall information to meet consumers’ actual needs is a constraint under which consumers choose unsafe food recall information and information composition in the options box. Information fitness was divided into five levels between 0 and 100% by the degree of fitness, such that the higher the information fitness level, the more complete the consumer’s unsafe food recall information needs. Consumers may choose whether to refer or not to refer, and the option “I choose neither” is included to provide a real choice environment. In this study, the Alternative Specific Constant (*ASC*), which indicates the strength of consumer preference for unsafe food recall information, was used to replace the constant term when processing the sample.

**Figure 4:**
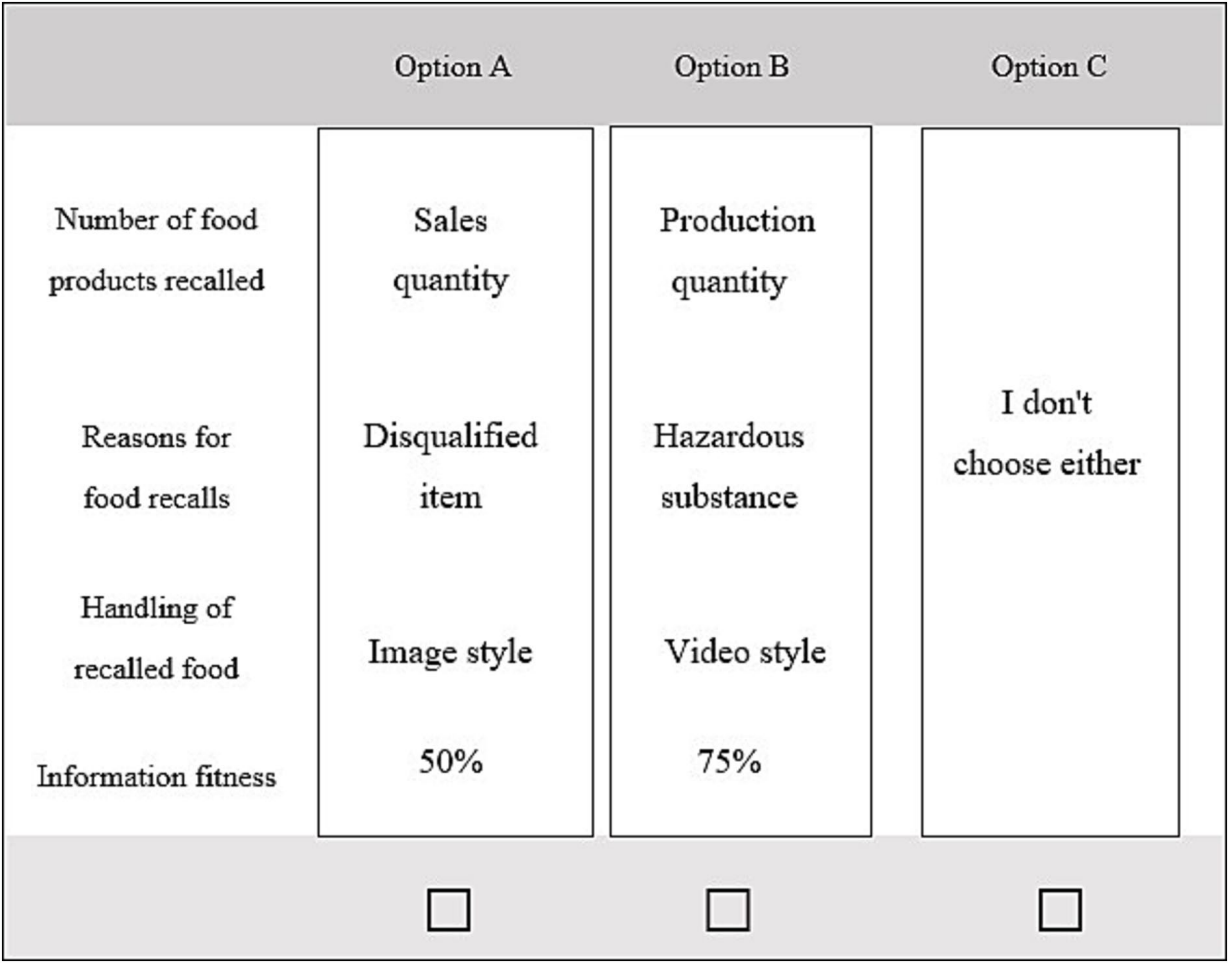
Example of a choice set for food recall information.

Full factorial designs can result in consumers being unable to answer a large number of choice sets; therefore, it is more appropriate to use partial factorial designs (84). In this study, an orthogonal design was constructed using D-efficiency, and a stata17 design was used to generate eight choice sets, each containing two alternative options and one *ASC* option. In the third stage of the pre-survey, 50 consumers were invited to comment on the design of the choice sets^2^ and the wording and description of the questions in the questionnaire, and corrections and adjustments were made accordingly. The questionnaire ensures that each choice set ultimately maintains a balance in the distribution of all levels for each attribute, that the combinations of different attributes satisfy balance, and that the utility of the alternatives in each choice set is approximately equal (84). To ensure that consumers understood all the available information when presented with the choice sets (Figure 4), explanatory appendices were compiled, providing detailed explanations of each attribute and the specific meanings represented by each level. For instance, under “*Hazardous substance*,” we present information such as “Benzoic acid and its sodium salt are relatively safe; a small amount of benzoic acid is non-toxic to the human body and can be excreted through urine. Prolonged excessive intake of food with excessive benzoic acid may have a certain impact on liver function.” Under “*Disqualified item*,” we display inspection results of a certain soy product, indicating “Disqualified item ║Inspection results║ standard value → Benzoic acid and its sodium salt (as benzoic acid)║1.34 g/kg║ not to be used.” For food recall handling, we downloaded historical data from official websites and media for presentation purposes.

### 3.4 Empirical model

The general principle of the Discrete Choice Model (*DCM*) is based on Lancaster’s (85) random utility theory, which posits that the utility of a good does not come from itself but from the utility attributes that it possesses, and consumers’ preferences determine the utility, which in turn affects their payment behavior (86). Therefore, information is an important commodity. There is variability in the utility that consumers derive from similar goods with different attributes. Conventional food safety certification information is optional, and consumers have the right to make their own choices of references when making purchase decisions. However, food companies are obligated to inform consumers when unsafe food is detected under China’s recall system, and consumers will maximize their own safety utility in the process of information transfer.

The Discrete Choice Model, based on Luce’s (87) Independence of Irrelevant Alternatives (*IIA*) assumption, defines the available profiles of unsafe food recall information, denoted as choice set *A*, as having *J* options. Let the $n_{th}\;$ consumer in scenario *t* choose the $i_{th}$ information profile from choice set *A* to obtain the utility $U_{nit}$ . The total utility of the consumer is stochastic and can be divided into deterministic utility $V_{nit}$ and random utility $\varepsilon_{nit}$ , expressed as Equation 8:


(8) $$U_{nit}=V_{nit}+\varepsilon_{nit},i\in A.$$

When the utility obtained by the $n_{th}\;$ consumer from unsafe food recall information profile *i* is greater than the utility obtained from another unsafe food recall information profile *j* (*j*∈*A* and *j* ≠ *i*), the consumer will choose unsafe food recall information profile *i*. The probability of a consumer selecting information profile *i* in scenario *t* is represented as the probability formula is expressed as Equation 9:


(9) $$P_{nit}=Prob\left(V_{nit}+\varepsilon_{nit}>V_{njt}+\varepsilon_{njt}\right),i,j\in A\;\mathrm{and}\;j\neq i\;.$$

In this study, the deterministic utility $V_{nit}$ is a function of the observed levels, referring to *Sales quantity*, *Stores quantity*, *Hazardous substance*, *Image style*, *Video style*, *Informatica fitness* (*IF*), and *ASC*. In Equation 10, the expression of $V_{nit}$ is as follows:


(10) $$\begin{array}{c} V_{nit}=ASC+\beta_{1}\cdot IF_{nit}+\beta_{2}\cdot Sales_{nit}+\beta_{3}\cdot Store_{nit}\\ +\beta_{4}\cdot Hazardous_{nit}+\beta_{5}\cdot Image_{nit}+\beta_{6}\cdot Video_{nit} \end{array}$$

The assumption that individual consumers have identical preferences often does not reflect reality. Assuming heterogeneity in consumer preferences, in the deterministic part of utility $V_{nit}$ = $\beta^{\prime }X_{nit}$ , where the random vector $\beta^{\prime }$ represents random coefficients following a certain distribution with probability density *f*(*β*), then the probability of a consumer choosing information profile *i* is given by Equation 11:


(11) $$P_{nit}=\int \frac{\exp\left(\beta^{\prime }X_{nit}\right)}{\sum_{j}^{J}\exp\left(\beta^{\prime }X_{njt}\right)}f\left(\beta \right)d\beta_{n}$$

The above equation represents the mixed logit model (MLM), which allows the coefficients of the explanatory variables to be random, thus overcoming the independence of irrelevant alternative (*IIA*) assumptions (88). The estimated coefficients of this model cannot be directly interpreted in terms of magnitude but rather by the significance and sign of the parameters, indicating preferences relative to the baseline level.

Willingness To Pay (*WTP*) in this study is defined as the extent to which consumers fit to unsafe food recall information disclosure policies, which is expressed by Equation 12:


(12) $$WTP_{ki}=\frac{E\left(\beta_{ki}\right)}{\beta_{IF}}$$

Relative Importance Value (*RIV*) is calculated to measure the relative importance of a certain information attribute within all available levels of unsafe food recall information profiles, indicating the degree of preference consumers have toward that attribute. The relative importance of information attribute *k* is the result of standardizing the difference between the maximum and minimum coefficients of that factor across the different levels, which is expressed by Equation 13:


(13) $$RIV_{k}=\frac{\max\left(\beta_{k}\right)-\min\left(\beta_{k}\right)}{\sum_{k}^{K}\left[\max\left(\beta_{k}\right)-\min\left(\beta_{k}\right)\right]}$$

## 4 Results and discussion

### 4.1 Consumers have cognitive dissonance

In this study, consumer emphasis on food quality was categorized based on their scores. A score of 0 was defined as “*No emphasis*” (percentage is 15.45%); scores of 1, 2, and 3 were grouped as “*Low emphasis*” (percentage is 16.53%); scores of 4, 5, and 6 as “*General emphasis*” (percentage is 26.34%); and scores of 7, 8, and 9 as “*High emphasis*” (percentage is 41.68%). Figure 2 illustrates the level of understanding of food safety-related information among consumers with different levels of emphasis on food quality in the sample. Across the entire sample, the understanding levels were as follows: Level = 0 (31.74 to 40.38%), Level = 1 (31.95 to 38.48%), Level = 2 (21.38 to 31.14%), and Level = 3 (1.80 to 3.56%). Furthermore, from a statistical perspective, the correlation coefficient between these two variables, tested using Stata17 software, was 0.007, with a *p*-value of 0.269, indicating a very low correlation. This suggests that consumers’ emphasis on quality does not align with their understanding of food safety information, leading to cognitive dissonance. Thus, Hypothesis 1 is validated. Next, we examined consumers’ actual cognition and preferences regarding unsafe food recall information, as well as the differences in information preferences among different groups.

### 4.2 Consumer preference for unsafe food recall information

Table 3 presents the consumer preference results for unsafe food recall information. In this study, *ASC* and *information fitness* (*IF*) were set as fixed parameters, and the other level variables were set as random parameters.

**Table 3:** Estimates for all samples.

Variables	(1)		(2)
	Mean	*SD*	Mean
Fixed parameter			
ASC	−0.735*** (0.093)		−0.809*** (0.088)
Information fitness(IF)	0.275*** (0.082)		0.225*** (0.077)
Random parameter			
Number of food products recalled			
Sales quantity	0.157*** (0.056)	0.295*** (0.095)	0.139*** (0.051)
Store quantity	0.428*** (0.049)	0.292*** (0.080)	0.354*** (0.043)
Reasons for food recalls			
Hazardous substance	0.656*** (0.049)	1.004*** (0.049)	0.563*** (0.033)
Handling of recalled food			
Image style	0.304*** (0.052)	−0.005 (0.145)	0.246*** (0.049)
Video style	0.248*** (0.039)	0.291*** (0.079)	0.218*** (0.035)
*N*	24,240		24,240
Log likelihood	−7176.3021		−7349.0990
LR χ^2^(5)	345.59		3055.38
Prob > χ^2^	<0.001		<0.001

*, **, and *** denote significance at the 10, 5, and 1% levels, respectively.

Column (1) indicates that the mixed logit model estimated all fixed parameters to be statistically significant at the 1% level. The coefficient of *ASC* is negative, suggesting that the utility derived from selecting nothing (Option C) is negative, whereas consumers derive positive utility from selecting information combinations (Options A and B). The significance of *IF* at the 1% level suggests that consumers tend to choose information combinations with higher degrees of fit, indicating that a higher degree of fit results in higher utility. Thus, Hypothesis 2a is validated. All random parameters have significant means at the 1% level, indicating significant preferences for the overall sample. The standard deviation of the “*Image style*” variable in the table is not significant, indicating no significant preference compared to the “*Text style*.” Compared to “*Production quantity*,” Chinese consumers are most concerned about the quantity of unsafe food in stores relevant to them, followed by the quantity of unsafe food circulating in the market. This finding suggests that consumers prioritize self-interest over concern about the adverse effects of unsafe food on society, which reflects a sense of altruism. Compared with the specific quantity values annotated by professionals for unqualified items, consumers significantly prefer an indication of the degree of harm to the human body caused by “*Hazardous Substances*,” even though such descriptions are vague for non-professionals (60). In terms of presenting unsafe food handling, consumers are more inclined to “*Video style*” than “*Text style.*” Similarly, Yu et al. (61) argued that innovative new media can effectively convey information visually compared with traditional media. In our interviews, we observed notable regional differences among Chinese consumers in their demand for and perception of recall information. These variations appear to be influenced by regional, cultural, educational, and economic factors. In the eastern region, characterized by international openness, strong interactions between economic and educational development enhance consumer awareness beyond personal interests. Consumers in this region often consider public health-related recall of information alongside individual concerns. In contrast, the central region, an inland area with a more traditional and self-centered cultural orientation, tends to focus primarily on the health hazards of recalled food. This focus may stem from relatively lower levels of economic and educational development compared to the eastern region. Zhang (89) explored this relationship, emphasizing that groups in wealthier regions tend to pay greater attention to food safety issues. Meanwhile, the western region, known for its diverse ethnic composition, exhibits significant individual and cultural differences. Geographic, political, and cultural boundaries in this area lead consumers to prioritize recall information that is directly relevant to their personal circumstances. This finding aligns with the conclusions of Zhou and Liu (90), who argued that food safety in China’s ethnic regions, especially in rural areas, is characterized by complexity, cross-boundary issues, and fragmentation. In addition, this study uses a conditional logit model as a robustness check. Column (2) shows that its results are fairly consistent with our other results, indicating that the estimation results are robust.

Table 4 presents the *RIV* and *WTP* for overall consumer estimation. In terms of *RIV*, the rankings are as follows: “*Reasons for food recalls*” (47.26%), followed by “*Number of food products recalled*” (30.83%) and “*Handling of recalled food*” (21.90%). Without considering *IF*, consumers are most concerned about the reasons for problematic food being deemed non-compliant or harmful to human health, followed by concerns about the quantity of unsafe food and whether it poses a threat to personal health or public safety, and lastly, concerns about how the government orders companies to handle recalled unsafe food, including issues such as secondary sales or recycling for further processing. Regarding *WTP*, consumers have the highest surplus for “*Hazardous substance*,” followed by “*Store quantity*” and “*Video style*,” indicating that the disclosure of information of these types best satisfies consumers’ cognition and preferences. The results show that consumers prefer unsafe food recall information, which reduces their search costs and lowers their cognitive costs. Thus, Hypothesis 2b is validated.

**Table 4:** RIV for Attributes and WTP for Levels.

Attributes	RIV	Attributes	WTP	95% confidence Intervals
Number of food products recalled	30.83%	Sales quantity	56.91%	[0.195, 0.943]
		Store quantity	155.52%	[0.790, 2.320]
Reasons for food recalls	47.26%	Hazardous substance	238.60%	[1.022, 3.750]
Handling of recalled food	21.90%	Image style	110.37%	[0.465, 1.742]
		Video style	90.04%	[0.265, 1.535]

### 4.3 Impact of past experiences with purchasing unsafe food on preferences for unsafe food recall information

Columns (3) and (4) in Table 5 show that consumers without past experience purchasing unsafe food do not prioritize information fitness. Those with such experiences prefer “*Store quantity*,” indicating that past experiences increase consumers’ concern about information related to themselves. Regarding the reasons for food recall, there was little difference in the preferences between the two groups. However, consumers with past experience clearly showed greater interest in how recalled unsafe food was handled, and they preferred to receive information in video formats. By contrast, consumers without such experiences showed no clear preferences; they said that how they handled the recalled unsafe food was of little concern to them and that the manner in which they communicated the recall handling information made little difference, as they would not care (insignificant standard deviation). These results were consistent with those of previous studies (62–64). This difference may be attributed to the “Bystander effect.” Thus, Hypothesis 3a is validated.

**Table 5:** Estimates for consumers’ past experience with unsafe food.

Variables	(3) Experience of buying unsafe food		(4) No experience of buying unsafe food	
	Mean	*SD*	Mean	*SD*
Fixed parameter				
ASC	−0.709*** (0.126)		−0.768*** (0.140)	
Information fitness(IF)	0.372*** (0.110)		0.152 (0.124)	
Random parameter				
Number of food products recalled				
Sales quantity	0.172** (0.075)	−0.117 (0.279)	0.135 (0.086)	0.420*** (0.108)
Store quantity	0.460*** (0.066)	0.321*** (0.100)	0.389*** (0.073)	0.284** (0.124)
Reasons for food recalls				
Hazardous substance	0.661*** (0.066)	1.029*** (0.066)	0.655*** (0.072)	0.980*** (0.073)
Handling of recalled food				
Image style	0.354*** (0.070)	0.024 (0.214)	0.240*** (0.079)	−0.020 (0.199)
Video style	0.237*** (0.054)	0.395*** (0.089)	0.261*** (0.058)	−0.023 (0.348)
*N*	13,584		10,656	
Log likelihood	−3986.7594		−3184.4087	
LR χ^2^(5)	207.69		142.50	
Prob > χ^2^	<0.001		<0.001	

*, **, and *** denote significance at the 10, 5, and 1% levels, respectively.

### 4.4 Composition of household members impacts preferences for unsafe food recall information

Columns (5) and (6) in Table 6 present the impact of the presence of pregnant women or children in Chinese households on the preferences for unsafe food recall information. The results indicate that households with pregnant women or children tend to prefer “*Sales quantity*,” “*Hazardous Substances*,” and “*Video style*.” By communicating with consumers, we identified several reasons for this preference. The presence of pregnant women or children in households prompts them to consider more factors because they are concerned that manufacturers may resell recalled unsafe food after processing it. Additionally, they worry about the possibility of unsafe food from other regions entering the local markets. During these discussions, we found that Chinese households with pregnant women or children tended to exhibit more empathy. They did not want other children or pregnant women to consume unsafe food, demonstrating a certain level of empathy-driven altruism. Conversely, consumers in households without pregnant women or children were more concerned about the presence of unsafe food in their local areas, and showed no significant interest in or preference for information on the handling of recalled unsafe food (insignificant standard deviation). This suggests that they are not concerned about how the recalled unsafe food is handled or whether it is resold or processed again. Previous studies reported similar findings (70, 71, 75). Thus, Hypothesis 3b is validated.

**Table 6:** Estimates for the presence of pregnant women or children in the household.

Variables	(5) Have pregnant women or children under 12 years old		(6) No pregnant women or children under 12 years old	
	Mean	*SD*	Mean	*SD*
Fixed parameter				
ASC	−0.563*** (0.167)		−0.809*** (0.113)	
Information fitness(IF)	0.373** (0.146)		0.233** (0.099)	
Random parameter				
Number of food products recalled				
Sales quantity	0.311*** (0.102)	0.288* (0.169)	0.088 (0.068)	−0.288** (0.119)
Store quantity	0.550*** (0.088)	−0.224 (0.169)	0.375*** (0.059)	0.339*** (0.087)
Reasons for food recalls				
Hazardous substance	0.719*** (0.084)	0.958*** (0.086)	0.626*** (0.059)	1.030*** (0.060)
Handling of recalled food				
Image style	0.257*** (0.092)	−0.002 (0.184)	0.325*** (0.064)	−0.006 (0.256)
Video style	0.269*** (0.072)	0.431*** (0.110)	0.238*** (0.047)	−0.200 (0.126)
*N*	7,800		16,440	
Log likelihood	−2288.0311		−4884.3724	
LR χ^2^(5)	97.10		248.80	
Prob > χ^2^	<0.001		<0.001	

*, **, and *** denote significance at the 10, 5, and 1% levels, respectively.

### 4.5 Influence of education level on preferences for unsafe food recall information

Chinese consumers who agreed to be interviewed were divided into two groups based on whether they had received education at the university or college level. Columns (7) and (8) of Table 7 show that consumers with higher education levels experience increased utility from *IF* improvement, whereas those without higher education do not. Higher education led consumers to pay more attention to “*Store quantity*” information regarding food recalls, indicating a stronger awareness of food safety risks, but they also paid attention to “*Sales quantity*.” Upon inquiry, these individuals stated that they were concerned that unsafe foods circulating in the market might be accessed by older adults, children, or individuals with weakened immune systems. They also considered the possibility that these products would be sold at low prices, which could harm financially disadvantaged individuals. Conversely, consumers without higher education only focused on “*Store quantity*,” their main concern being whether they or their family members had consumed unsafe food, showing no significant preference for “*Sales quantity*.” This suggests that higher education can enhance cognitive empathy among Chinese residents. Consumers with higher education showed a greater preference for video presentations of recall handling information, whereas those without higher education preferred image presentations. The latter group believed that it would take longer to process the information in videos, despite them containing more content than images, considering that images are simpler and more intuitive (91). Thus, Hypothesis 4 is validated.

**Table 7:** Estimates for education level.

Variables	(7) Received regular college or junior college education		(8) Did not receive regular college or junior college education	
	Mean	*SD*	Mean	*SD*
Fixed parameter				
ASC	−0.590*** (0.106)		−1.225*** (0.200)	
Information fitness (IF)	0.341*** (0.094)		0.060 (0.174)	
Random parameter				
Number of food products recalled				
Sales quantity	0.209*** (0.064)	0.267** (0.118)	−0.019 (0.120)	0.282 (0.216)
Store quantity	0.449*** (0.056)	0.303*** (0.091)	0.373*** (0.102)	0.316** (0.152)
Reasons for food recalls				
Hazardous substance	0.686*** (0.056)	1.038*** (0.056)	0.583*** (0.099)	0.887*** (0.104)
Handling of recalled food				
Image style	0.333*** (0.059)	0.017 (0.120)	0.202* (0.116)	0.430*** (0.162)
Video style	0.291*** (0.044)	0.206* (0.122)	0.100 (0.085)	0.422*** (0.134)
*N*	18,912		5,328	
Log likelihood	−5600.0054		−1563.1292	
LR χ^2^(5)	299.54		53.67	
Prob > χ^2^	<0.001		<0.001	

*, **, and *** denote significance at the 10, 5, and 1% levels, respectively.

## 5 Conclusion

From a theoretical perspective, government-led information dissemination is expected to benefit consumers (2). However, in practice, food recalls for unsafe products in China remain inefficient, with consumers often struggling to access complete recall information (3). This inefficiency reflects shortcomings in China’s food safety information governance policies. A key finding of this study is that one-size-fits-all risk communication policies have resulted in cognitive dissonance among consumers by failing to address their diverse information needs and perceptions. Furthermore, cultural, regional, individual, and household factors significantly influence consumer information requirements. By analyzing the heterogeneity of these needs, this study offers valuable insights to enhance government information governance efficiency, improve consumer understanding, reduce potential food safety risks, and support public health initiatives.

This study employs the search and cognitive cost theories to explain and validate consumer preferences for recalling information. Consumers prioritize information such as the reasons for food recalls, the number of food products recalled, and the handling of recalled food. They prefer information with high search costs, such as the “*Store quantity*,” and low cognitive costs, such as “*Harmful substances*” and “*Video style*.” While consumers tend to focus on information related to personal interests, collectivist values, and moral education also encourage altruistic awareness. For instance, they pay attention to public interest-related information, such as “*Sales quantity.*” These findings demonstrate that recall information enhances consumer utility.

This study also examined variations in recall information preferences among different consumer groups. Results revealed a strong preference across all groups for information about “*Hazardous substances*,” aligning with cognitive levels and interests. Groups that have experienced harm from unsafe food place greater emphasis on “*Store quantity*” and “*Handling of recalled food*,” whereas those without such experiences show less interest in recall process details. This “bystander effect” implies that consumers who have experienced harm are more vigilant about recall information and are more inclined to engage with public health-related information. Moreover, households with pregnant women and children demonstrated stronger preferences for recall information. They not only focus on the “*Store quantity*” but also pay attention to the “*Sales quantity*” of affected food in the market and whether “*Handling of recalled food*” could cause secondary harm to society. In contrast, households without pregnant women or children primarily prefer information about “*Store quantity*.” Influenced by traditional cultures and emotional empathy, mothers exhibit a strong sense of collective identity and altruism.

The role of education in recall information cognition was also explored. Results show that individuals with higher education levels not only prefer the “*Store quantity*” but also pay attention to public health-related information, such as “*Sales quantity*.” whereas less-educated groups focus solely on “*Store quantity*.” This indicates that education positively influences moral awareness and public-interest cognition, fostering altruistic behavior through enhanced cognitive empathy. Moreover, individuals with higher education levels prefer “*Video style*” information presentation, while those without higher education prefer “*Image style*” formats. This finding suggests that higher education enables consumers to comprehend complex new media formats. Therefore, continuously improving education levels can enhance public participation in food safety information governance and improve the ability to process new media information.

## 6 Policy implications

Based on these findings, we recommend that the Chinese government enhance the systematic and comprehensive disclosure of unsafe food recall information. Improving the transparency and clarity of such disclosures can help consumers better understand the specific reasons for recalls, the scope of affected products, potential health risks, and necessary actions. This would reduce consumers’ cognitive and search costs while preventing cognitive dissonance. To address the diverse and complex demands for food safety information, governments can leverage modern technologies such as big data and cloud computing to provide tailored and differentiated information and services for consumers. This approach not only strengthens consumers’ right to know but also builds public trust in food safety. In addition to reinforcing oversight of food safety, the government should encourage public participation in food safety monitoring. Increased societal awareness and engagement would contribute to an improved food safety governance system. Our findings, which reveal differing levels of attention to food recall information among various consumer groups, offer new perspectives for fostering societal empathy and compassion. Special attention should be placed on enhancing public food safety education, particularly for households with pregnant women or children, to increase their sensitivity and ability to assess food safety information. This would promote greater public health awareness and elevate health standards. As an open and globally integrated nation, China must align with international standards while considering cross-national differences in addressing the realities of transnational population flows. The government should ensure the complete disclosure of food recall information to foreign residents in China as well as to export destination countries for Chinese goods, thereby upholding its global public health responsibilities.

## 7 Limitations and future research

This study had several limitations. First, while it examines the role of social media in disclosing unsafe food recall information, it does not further analyze the impact of different social media platforms, such as WeChat groups or TikTok, on consumers’ information needs and perceptions. Second, this study focused on aligning the content of information disclosure with consumer needs and perceptions. However, a more comprehensive comparison of dimensions, such as the medium, method, and content of information disclosure, should also be conducted. This would help governments allocate resources and prioritize efforts more effectively across channels. Finally, explanations of regional and cultural differences in consumer perceptions and preferences are primarily derived from qualitative insights obtained through interviews. These insights lack empirical analysis of how factors such as regional culture, economic development, and religious beliefs. Future research should further explore these areas.

## Data Availability

The original contributions presented in the study are included in the article/supplementary material, further inquiries can be directed to the corresponding author.

## Footnotes

^1^Data were collected from the “Risk Control” section of the official website of the Market Supervision and Administration of the 31 provinces of mainland China. The author excluded catering food and edible produce, calculating each province’s processed food recall rate as the arithmetic average of the province’s recall rate for each batch of problem food in that year. The national recall rate for processed foods is the arithmetic mean of the recall rates for all provinces for the year, with missing values for some provinces, and the resulting data are approximate ranges. Notes: Recall rate per batch = actual recalls/quantity sold.

^2^In the third stage of the pre-survey process, consumers indicated that options with 0% information fitness and certain level combinations in the initial computer-generated choice set appeared too frequently. This caused sensory discomfort and made them more likely to discard the 0% counterpart of the alternative option during the choice process. Therefore, to prevent the alternative option from being discarded due to external factors, this study avoided the 0% information fitness scenario in the formal study. Additionally, the high frequency of certain combinations in the initial choice set caused respondents to feel bored, making them prefer the *ASC* option or another alternative. As a result, the choice set was adjusted in the formal questionnaire.
